# Single nucleotide polymorphism rs3774261 in the AdipoQ gene is associated with the risk of coronary heart disease (CHD) in Northeast Han Chinese population: a case-control study

**DOI:** 10.1186/s12944-015-0173-4

**Published:** 2016-01-12

**Authors:** Joseph Sam Kanu, Yulu Gu, Sun Zhi, Mingxi Yu, Yuping Lu, Yetong Cong, Yunkai Liu, Yong Li, Yaqin Yu, Yi Cheng, Yawen Liu

**Affiliations:** Department of Epidemiology and Biostatistics, School of Public Health of Jilin University, Changchun, 130021 China; Clinical laboratory of China-Japan Union Hospital of Jilin University, Changchun, China; The General Hospital of Jilin Chemical Group Corporation, Jilin, 132022 China; The Cardiovascular Center, the First Hospital of Jilin University, Changchun, 130021 China

**Keywords:** AdipoQ, Adiponectin, Polymorphism, Coronary heart disease, Risk, Association, Interaction

## Abstract

**Background:**

Coronary Heart Disease (CHD) is one of the leading causes of death in the world with a projected global 82 million DALYs by 2020. Genetic and environmental factors contribute to CHD development. Here, the authors investigate the association between CHD risk and three Single Nucleotide Polymorphisms (SNPs) in the AdipoQ gene (rs3774261, rs1063537 and rs2082940); and the interaction of this association with environmental factors, in Northeast Han Chinese population.

**Methods:**

Using a case–control study design, 1514 participants (754 cases and 760 controls) were investigated. Three variants in the AdipoQ gene (rs3774261, rs1063537 and rs2082940) were selected and genotyped. The online SNPstats program and SPSS 21.0 software were used for data analyses.

**Results:**

The authors found that the rs3774261G allele is associated with the risk of CHD but that the rs2082940T allele protects against CHD. No significant association was found between rs1063537 and CHD risk. The study also found significant interactions between triglyceride levels and the SNPs studied (*P* < 0.0001 for rs3774261, *P* = 0.014 for rs1063537, and *P* = 0.031 for rs2082940).

**Conclusions:**

Variations in AdipoQ gene can protect against CHD (as with rs2082940T) or associated with CHD risk (as with rs3774261G) in Northeast Han Chinese – findings that will help shed light on the reported conflicting roles of AdipoQ in cardiovascular diseases. Serum triglycerides levels also interact in the AdipoQ – CHD association, thus further highlighting the roles environmental factors play in the genetic aspect of diseases.

## Background

Coronary Heart Disease [also known as Coronary Artery Disease (CAD), Ischemic Heart Disease (IHD), Atherosclerotic Heart Disease, or Atherosclerotic Cardiovascular Disease], is a group of diseases that includes: stable angina, unstable angina, myocardial infarction, and sudden coronary death [1]. The challenges (mortality and disability) posed by CHD are not only crucial to the industrialized countries but also remain public health problems for developing countries [2–4]. In 2013 CHD was the most common cause of death globally, resulting in 8.14 million deaths (16.8 %) up from 5.74 million deaths (12 %) in 1990 [5]. Coronary heart disease burden is projected to rise from around 47 million DALYs globally in 1990 to 82 million DALYs in 2020 [6].

Mortality from CHD in the Chinese population is relatively low (when compared with Western countries) but its burden has been increasing and reported to be the second leading cause of death from cardiovascular diseases [7], accounting for 22 and 13 % of cardiovascular deaths in urban and rural areas of China respectively [8].

Although coronary atherosclerosis (plaque formation on heart vessels mainly due to fats buildup and inflammation, leading to narrowing of the vessel lumen and decreased blood flow to heart muscles), is generally considered the pathological foundation of CHD [9–11], the exact causes and pathogenesis of this disease are not fully understood. However, several studies have shown that CHD is a complex disease that is caused by many factors (including environmental, lifestyle and genetic predisposition), and the interactions occurring among these factors [12–20].

In a search for genes associated with coronary artery disease, Roberts and colleagues identified several thousand significant single nucleotide polymorphisms (SNPs) and 130 clusters associated with CAD [21]. Epidemiological and family studies have repeatedly shown that genetic predisposition accounts for 40 to 60 % of the risk for CAD [22]; and thus preventing and treating CAD in a comprehensive manner is expected to also include modifying the effects of these genetic risk factors, alongside the current treatment of hypercholesterolemia, which is partly genetic and partly environmental [21].

AdipoQ (Adiponectin, C1Q and collagen domain containing) gene, located on chromosome 3 (Map Location: 3q27) and expressed exclusively in adipose tissue, is among the genes associated with CHD. It consists of 3 exons and 2 introns spanning a 17-kb region [23]. Several single nucleotide polymorphisms (SNPs) have been found in the AdipoQ, including the rs3774261, rs1063537 and rs2082940, and many of these SNPs have been associated with various disease conditions such as CHD, diabetes, hypertension, obesity, rheumatoid arthritis, myocardial infarction, and many more [24–40]. Findings from previous studies on these AdipoQ gene polymorphisms in relation to CHD and other cardiovascular disease risks are not only inconsistent and inconclusive, but most of these studies are conducted on animal models [41, 42], Europeans [27, 31] or other Western populations [43, 44]. In addition, to the best of our knowledge, studies on Northeast Han Chinese populations investigating the genetic link between CHD risk and the rs3774261, rs1063537 and rs2082940 SNPs in the AdipoQ gene have not been reported.

Hence, the primary objective of this study was to investigate the genetic association between the risk of CHD and the rs3774261, rs1063537 and rs2082940 SNPs of AdipoQ gene; and also search for possible interactions of these polymorphisms with environmental factors in the susceptibility to CHD among Northeast Han Chinese populations.

## Methods

### Study participants

This study included 1514 participants: 754 CHD patients and 760 controls, enrolled in a case–control study design. The CHD patients were recruited from two separate sources: The First Norman Bethune Hospital of Jilin University in Changchun, and The PetroChina Jilin General Hospital – both sources located in the North-eastern province of Jilin in China. The controls, matched for age and sex, were randomly recruited during routine health examination at the sources mentioned above, at the same time as the cases were recruited. At least two experienced cardiologists were needed for the diagnosis of CHD and the diagnosis confirmed by coronary angiography (>50 % diameter stenosis in at least one of the major coronary arteries) according to the World Health Organization criteria for the confirmation of CHD. Electrocardiographic profile and clinical evaluations were used to determine that the controls were CHD free. All controls had no history of diabetes and hypertension. Both the CHD cases and controls were unrelated Han Chinese population in North-east China.

The study was approved by the Ethics Committee of the School of Public Health, Jilin University, Changchun; and informed consent obtained from all participants included in the study.

### Epidemiological survey and biochemical examination

A structured questionnaire was used to obtain participants’ demographic information and clinical characteristics. A smoker was defined as a person who smoked at least one cigarette per day in the past 1 month, or a past smoker even if the participant had completely abstained from cigarette use for at least 1 month. An alcoholic drinker was defined as a person who consumed any kind of alcoholic beverage on average more than once a week. We obtained samples of fasting blood from all participants. MODULE P800 automated biochemistry analyzer (ROCHE, USA) was used to measure plasma lipid concentrations of all participants. Total cholesterol (TC) and serum triglycerides (TG), high density lipoprotein cholesterol (HDL-C) and low density lipoprotein cholesterol (LDL-C) profiles from both cases and controls were measured.

### SNP selection

The HapMap website and Haploview 4.2 software (Daly Lab. at the Broad Institute, USA) were used to select three SNPs in the AdipoQ gene. The rs1063537 and rs2082940 SNPs reside in the 3’UTR and the rs3774261 resides in intron 2 of the AdipoQ gene. The minor allele frequencies (MAF) of the three studied SNPs were all greater than 5 %.

### DNA extraction

The blood specimens from each participant were placed in non-anticoagulant, plexiglass tubes and stored at -20 °C until DNA extraction. Genomic DNA was extracted using a blood DNA extraction kit (ClotBlood DNA kit, Cwbio, Beijing, China) as specified in the manufacturer’s instructions. The purity and concentration of the extracted DNA were checked using ultraviolet spectrophotometer (Beckman, USA) with ultraviolet (UV) readings at 260 nm and 280 nm, respectively. The extracted DNA was then stored at 4 °C for further analysis

### SNP genotyping

Standard polymerase chain reaction (PCR) protocols were used to amplify the DNA specimens. Assay Design 3.1 software (Sequenom Inc., San Diego, CA, USA) was used to design the Primers for the PCR process. The PCR reaction was done in a total volume of 5 μL containing 1 μL DNA sample (10 ng/ μL), MgCl_2_ 1.625 mM, 500 μM dNTP, PCR Buffer 1×, 1 unit HotStart Taq DNA polymerase, 0.1 μM PCR primers, and 1.8 μL ddH_2_O. The PCR cycling started with an initial denaturation at 94 °C for 15 min, followed by 45 cycles of denaturation at 94 °C for 20 s, annealing at 56 °C for 30 s and extension at 72 °C for 1 min, and a final extension at 72 °C for 3 min. SNP genotyping was determined by Matrix-Assisted Laser Desorption/Ionization Time of Flight Mass Spectrometry (MALDI-TOF-MS) in the MassARRAY system (Sequenom, San, Diego, CA, USA). PCR reaction was performed on 384-well plates using a MassARRAY Nanodispenser (Sequenom). As quality control measures, the extracted DNA was checked for purity and concentration using ultraviolet spectrophotometer at ultraviolet (UV) readings of 260 nm and 280 nm (Beckman, USA). This was then followed by electrophoresis, PCR amplification, and cluster analysis with samples showing good scatter plots accepted for the final genotyping. Both blinded and unblinded samples were tested separately and replicated concordance rates of >99.99 %.

### Statistical analyses

Categorical variables were presented as absolute values and percentages, while Continuous variables were expressed as mean ± standard deviation (SD). Student’s t test was used to compare continuous variables, while Chi-square test was used to compare categorical variables between cases and controls. Chi-square test was also used for the Hardy-Weinberg equilibrium (HWE) test [45] and to analyse the genotype distributions of each SNP. We used analysis of covariance (ANCOVA) to assess the effect of the different genotypes and alleles on plasma lipid levels. Associations between AdipoQ polymorphisms and the risk of CHD were evaluated using multiple logistic regression analyses. Age, sex, cigarette smoking, and alcohol drinking were used as covariates in both the ANCOVA and multiple logistic regression analyses. Different genetic models (codominant, dominant, recessive, overdominant and log-additive) were used to evaluate the risk of CHD associated with each SNP. The Akaike information criterion (AIC) was used to determine the best model of inheritance for each SNP. Bonferroni correction was applied for multiple testing to reduce TypeIerror in association analysis. Linkage disequilibrium (LD) between SNPs was examined by Dʹ and r^2^. Interaction between AdipoQ gene polymorphisms and plasma lipids in relation to the risk of CHD was estimated by multiple logistic regressions. All statistical analyses were performed using SPSS 21.0 software and the online SNPStats program [46]. Two-sided test with *P* < 0.05 was considered statistically significant.

## Results

Table 1 summarizes the participants’ demographic data, clinical characteristics, and plasma lipids. Out of the 1514 genotyped DNA samples, 754 were from CHD patients and 760 from controls. No significant difference in age and sex between CHD patients and controls (*P* > 0.05) was detected, suggesting well matched participants for age and sex. CHD cases had higher levels of TC, TG, and LDL-C, but lower HDL-C levels than controls. The CHD group had more alcohol drinkers and cigarette smokers than the control group (14.2 % vs. 7.2 % for drinkers, 50.1 % vs. 11.6 % for smokers). The prevalence of hypertension and diabetes mellitus was also higher in CHD patients than controls (all *P*-values < 0.001).

**Table 1:** Demographics and lipid profile of participants (Northeast Han Chinese)

Variable	CHD (*n* = 754)	Controls (*n* = 760)	P*
Sex (M/F)	404/350	378/382	0.14
Age (years)	63.12 ± 8.05	63.10 ± 8.37	0.96
Diabetes mellitus, n (%)	110(7.3)	0(0.0)	<0.001
SBP(mmHg)	158.21 ± 34.45	124.27 ± 12.48	<0.001
DBP(mmHg)	92.60 ± 18.61	81.44 ± 38.87	<0.001
Hypertension, n (%)	444(58.9)	0(0.0)	<0.001
Alcohol drinking, n(%)	107 (14.2)	55 (7.2)	<0.001
Smoking, n(%)	378 (50.1)	88 (11.6)	<0.001
LDL-C (mmol/L)	2.78 ± 0.89	2.62 ± 0.71	<0.001
HDL-C (mmol/L)	1.15 ± 0.33	1.31 ± 0.25	<0.001
TG (mmol/L)	1.73 ± 1.12	1.25 ± 0.66	<0.001
TC (mmol/L)	4.35 ± 1.06	4.11 ± 0.80	<0.001

*TG* triglyceride, *TC* total cholesterol, *LDL-C* low-density lipoprotein cholesterol, *HDL-C* high-density lipoprotein cholesterol

*Analyzed by Student’s t test for continuous variable and by Pearson’s chi-square test for categorical variable

Table 2 shows the positions of the three SNPs, major and minor alleles, MAF, genotyping rate and the results of the HWE test of the studied SNPs. No deviation from the HWE in genotype distributions of all SNPs among controls was found (*P* > 0.05). Unlike SNP rs3774261 which is intron located, SNPs rs1063537 and rs2082940 are 3’UTR located on the AdipoQ gene.

**Table 2:** Information on the selected AdipoQ gene SNPs

NCBI SNP ID	Gene position	Alleles (major/minor)	MAF (case/control)	%Geno	*P* value of HWE in controls
rs3774261	Intron 2	A/G	0.49/ 0.41	98.88	0.20
rs1063537	3’UTR	C/T	0.26/ 0.28	99.01	0.42
rs2082940	3’UTR	C/T	0.26/ 0.30	98.88	0.60

*UTR* untranslated region, *MAF* minor allele frequency, *HWE* Hardy-Weinberg equilibrium. rs3774261 (+349 A > G), rs1063537 (+3228 C > T), rs2082940 (+3317 T > C)

%Geno is the percentage of non-missing genotypes for each marker

We evaluated the effects of different AdipoQ gene genotypes and alleles on plasma lipids in control subjects and the results are shown in Table 3. None of the studied SNPs showed significant association with plasma lipids level among controls (*P* > 0.05).

**Table 3:** Lipid profiles in different carriers of ADIPOQ genotypes and alleles in controls

Variable	No.	TG (mmol/L)	TC (mmol/L)	LDL-C (mmol/L)	HDL-C (mmol/L)
rs3774261	n				
AA	274	1.21 ± 0.67	4.11 ± 0.81	2.65 ± 0.71	1.30 ± 0.24
AG	350	1.27 ± 0.68	4.14 ± 0.81	2.63 ± 0.74	1.32 ± 0.25
GG	136	1.24 ± 0.59	4.04 ± 0.78	2.55 ± 0.66	1.31 ± 0.26
*P* ^a^		0.513	0.330	0.424	0.572
AA + AG	486	1.27 ± 0.66	4.12 ± 0.80	2.61 ± 0.72	1.32 ± 0.26
*P* ^b^		0.292	0.669	0.517	0.323
rs1063537	n				
TT	56	1.34 ± 0.94	4.06 ± 0.65	2.55 ± 0.58	1.32 ± 0.27
CT	319	1.23 ± 0.65	4.13 ± 0.80	2.64 ± 0.72	1.31 ± 0.24
CC	385	1.25 ± 0.62	4.10 ± 0.83	2.62 ± 0.73	1.31 ± 0.26
*P* ^a^		0.521	0.767	0.684	0.966
TT + CT	375	1.24 ± 0.70	4.12 ± 0.78	2.63 ± 0.70	1.31 ± 0.24
*P* ^c^		0.850	0.769	0.914	0.932
rs2082940	n				
TT	63	1.32 ± 0.91	4.05 ± 0.62	2.55 ± 0.57	1.32 ± 0.27
CT	323	1.20 ± 0.60	4.11 ± 0.80	2.63 ± 0.73	1.31 ± 0.24
CC	374	1.28 ± 0.67	4.12 ± 0.84	2.63 ± 0.73	1.32 ± 0.26
*P* ^a^		0.195	0.785	0.663	0.953
TT + CT	386	1.22 ± 0.66	4.10 ± 0.77	2.62 ± 0.71	1.31 ± 0.25
*P* ^c^		0.235	0.717	0.831	0. 815

*P* ^a^, comparisons among all gennotypes; *P* ^b^, minor allele homozygote serves as the reference; *P* ^c^, major allele homozygote serves as the reference. *P* values were calculated with ANCOVA adjusted for age, and sex

Distributions of genotypes and allelic frequencies among participants are shown in Table 4. The genotype distribution (*χ*^*2*^ = 24.75, *P* < 0.001) and allele frequency (*χ*^2^ = 21.33, *P* < 0.001) of rs3774261 are significantly different between CHD patients and controls. The genotype distribution of rs2082940 in CHD cases and controls are significantly different (*χ*^2^ = 4. 36, *P =* 0.04), but no significant difference was found for rs2082940 allele frequency between CHD patients and controls (*χ*^2^ = 0.34, *P =* 0.562). Genotype distribution and allelic frequency of rs1063537 were similar among CHD cases and controls (*P* > 0.05).

**Table 4:** Genotypes distributions and allelic frequencies of the three SNPs among participants

SNP	CHD (%)	Controls (%)	χ ^2^	*P*
rs3774261 (*n* = 1497)				
AA	180 (0.24)	274 (0.36)	24.75	**<0.001**
AG	387 (0.53)	350 (0.46)		
GG	170 (0.23)	136 (0.18)		
G allele	727 (0.49)	622 (0.41)	21.33	**<0.001**
rs1063537 (*n* = 1499)				
TT	51 (0.07)	56 (0.07)	2.60	0. 27
CT	283 (0.38)	319 (0.42)		
CC	405 (0.55)	385 (0.51)		
T allele	385 (0.26)	431 (0.28)	2.01	0.16
rs2082940 (*n* = 1497)				
TT	51 (0.07)	63 (0.088)	4. 36	**0. 04**
CT	283 (0.38)	323 (0.42)		
CC	403 (0.55)	374 (0.49)		
T allele	449 (0.3)	385 (0.26)	0.34	0.562

Data were analyzed by Pearson’s chi-square test. Bold values are statistically significant

Table 5 shows the results of the analyses of the association between the three selected SNPs of the AdipoQ gene and the risk of CHD under different inheritance models. We found that the G allele (and AG and GG genotypes) of rs3774261 is associated with the risk of CHD under all the inheritance models studied (codominant, dominant, recessive, overdominant and Log-additive). The T allele of rs2082940 is protective against CHD but only statistically significant under the dominant and Log-additive models of inheritance (*P* values =0.035 and 0.04 respectively). No significant association was found between rs1063537 and CHD risk under all the models studied. After Bonferroni correction (Bonferroni correction value =0.008 (0.05/6 = 0.008), the association between rs3774261 and CHD remained significant whereas the protective effect of the rs2082940 T allele was no longer significant.

**Table 5:** Associations between AdipoQ gene polymorphisms and CHD risk

SNPs	Models	*OR*	*95%CI*	*P*	AIC
**rs3774261**	Codominant	**1.68**	**1.32–2.13**	**<0.0001**	**2058.1**
		**1.90**	**1.41–2.54**	**<0.0001**	
	Dominant	**1.74**	**1.39–2.18**	**<0.0001**	**2056.9**
	Recessive	**1.37**	**1.07–1.77**	**0.014**	**2074.6**
	Overdominant	**1.29**	**1.06–1.59**	**0.013**	**2074.5**
	Log-additive	**1.40**	**1.21–1.62**	**<0.0001**	**2059.7**
**rs1063537**	Codominant	0.84	0.68–1.04	0.112	2082.9
		0.88	0.59–1.32	0.531	
	Dominant	0.85	0.69–1.04	0.11	2080.9
	Recessive	0.95	0.64–1.40	0.78	2083.4
	Overdominant	0.85	0.70–1.05	0.14	2081.3
	Log-additive	0.89	0.76–1.05	0.16	2081.5
**rs2082940**	Codominant	0.81	0.66–1.00	0.056	2078.1
		0.76	0.51–1.13	0.179	
	Dominant	**0.80**	**0.66–0.99**	**0.035**	**2076.2**
	Recessive	0.83	0.57–1.23	0.360	2079.8
	Overdominant	0.84	0.68–1.03	0.1	2077.9
	Log-additive	**0.84**	**0.72–0.99**	**0.04**	**2076.4**

*OR* odds ratio, *CI* confidence interval, *AIC* akaike information criterion. Codominant model (homozygote for the major allele serves as the reference); Dominant model (minor allele homozygote + heterozygote vs. major allele homozygote); Recessive model (minor allele homozygote vs. major allele homozygote + heterozygote); Overdominant model (heterozygote GA vs homozygote AA + GG serve as reference); Log-additive model (compares the relative significance of the minor allele : 0,1,2 in AA, AG, and GG respectively for the G allele of rs3774261; and 0,1,2 in CC, CT, and TT respectively for the T allele of rs1063537 and rs2082940). Bold values are statistically significant.

Unconditional logistic regression was used for data analyses. Age and sex, were adjusted during analyses. Bonferroni correction value =0.008 (0.05/6 = 0.008)

Table 6 presents the results of the analyses of the Pairwise linkage disequilibrium (*Dʹ*) and correlation coefficient (*r*^*2*^) among the studied polymorphisms in the AdipoQ gene. We found strong linkage disequilibrium (LD) among the three polymorphisms studied.

**Table 6:** Pairwise linkage disequilibrium (*D’*) and correlation coefficient (*r* ^*2*^) between the three SNPs in the ADIPOQ gene

SNP	*Dʹ*	*r*	*r* ^*2*^
rs3774261 - rs1063537	0.9994	- 0.5534	0.3063
rs3774261 - rs2082940	0.9994	- 0.5624	0.3163
rs1063537 - rs2082940	0.9799	0.9644	0.9301

Haplotype analysis is shown in Table 7. Three haplotypes accounted for 100 % of CHD patients but four haplotypes accounted for 99.19 % of the controls. The GCC haplotype with the most frequent allele at each site was defined as the reference. The haplotypes ATT (OR = 0.78, 95%CI =0.65 - 0.93, *P* = 0.0068) and ACC (OR = 0.74, 95%CI = 0.62 - 0.88, *P* < 0.0001) were more frequent in the controls than CHD patients and found to significantly protect against the risk of CHD. The ACT haplotype was only identified among controls with a frequency of 1.99 % but none among the CHD patients.

**Table 7:** Haplotypes of AdipoQ Gene with the risk of CHD

	Haplotypes			Frequencies			OR (95%CI)	*P*
No.	rs3774261	rs1063537	rs2082940	Total	CHD	Controls		
1	G	C	C	0.4508	0.4937	0.4092	1.00	
2	A	T	T	0.2681	0.2605	0.2755	**0.78 (0.65 - 0.93)**	**0.0068**
3	A	C	C	0.2669	0.2458	0.2873	**0.74 (0.62 - 0.88)**	**7 × 10** ^**4**^
4	A	C	T	0.0101	0	0.0199		
Rare				0.0041	0	0.0018		

The analysis was adjusted by age and sex. Bold values are statistically significant. The reference group was the most common haplotype (GCC). Rare: haplotypes with frequencies below 0.01

We examined the possible interactions between each of the selected polymorphisms of the AdipoQ gene and plasma TG levels in modifying the risk of CHD, and results are presented in Table 8. We found significant interactions between TG levels and three polymorphisms (*P* < 0.0001 for rs3774261, *P* = 0.014 for rs1063537, and *P* = 0.031 for rs2082940) of the AdipoQ gene. Wild homozygotes of each polymorphism with plasma TG < 1.70 mmol/L were defined as reference groups. Across the three polymorphisms, study subjects with plasma TG in the range1.70 ≤ TG < 2.26 had the highest ORs to develop CHD. For each of the three polymorphisms, the OR for developing CHD was largest among subjects who were homozygous for the major allele except for the minor allele GG of the rs3774261 polymorphism with the highest OR of 19.68 (5.86-66.03). The ORs for developing CHD were not significant for the minor allele TT homozygous of rs2082940. In subjects with TG ≥ 2.26 mmol/L, the OR to develop CHD was greatest in those with the GG genotype of the rs3774261 polymorphism (OR = 14.87, 95 % CI =5.10-43.37).

**Table 8:** ORs to develop CHD with different genotypes of three polymorphisms and plasma triglycerides (TG) levels*

Lipid levels (mmol/L)	rs3774261			rs1063537			rs2082940		
	AA	AG	GG	CC	CT	TT	CC	CT	TT
TG < 1.70	1.00^a^	2.15 (1.64-2.83)	1.87 (1.34-2.62)	1.00^a^	0.91 (0.71-1.16)	0.75 (0.47-1.18)	1.00^a^	0.85 (0.66-1.08)	0.65 (0.41-1.02)
1.70 ≤ TG < 2.26	9.45 (4.04-22.08)	3.74 (2.30-6.08)	19.68 (5.86-66.03)	3.80 (2.21-6.51)	2.42 (1.43-4.09)	---	3.24 (1.92-5.48)	2.56 (1.49-4.39)	---
TG ≥ 2.26	4.55 (2.32-8.89)	2.59 (1.62-4.13)	14.87 (5.10-43.37)	2.91 (1.79-4.72)	1.44 (0.87-2.38)	4.86 (1.02-23.12)	2.70 (1.67-4.35)	1.48 (0.89-2.48)	3.10 (0.81-11.79)
P-value**	**<0.0001**			**0.014**			**0.031**		

*Interactions with other lipid profiles, diabetes, hypertension, smoking and alcohol drinking were not statistically significant (data not shown)

^a^This group was the reference group

**Interaction between each polymorphism and plasma TG levels; unconditional logistic regression adjusted for age and sex was used in calculating OR, 95 %CI, and *P*-values of interactions. Bold values are statistically significant

## Discussions

Our study aimed to investigate the genetic association between three separate SNPs of AdipoQ gene (rs3774261, rs1063537and rs2082940) and Coronary heart disease (CHD), and the interactions of this association with certain variables (lipid profiles, diabetes, hypertension, smoking and alcohol drinking). The results revealed that CHD cases had higher levels of TC, TG, and LDL-C, but lower HDL-C levels than controls. The prevalence of hypertension and diabetes mellitus was also higher in CHD patients than controls (all *P* values <0.001); and the CHD group had more alcohol drinkers and cigarette smokers than the control group. The genotypes distributions of the rs3774261 and rs2082940 SNPs were significantly different between CHD cases and controls, but that of rs1063537 was the same in both groups. Association analyses under different inheritance models revealed that rs3774261G is associated with the risk of CHD while rs2082940T is protective against CHD, with SNP rs1063537T showing no significant association with CHD risk in our study population. The haplotypes ATT and ACC were more frequent in the controls than the CHD patients and is found to significantly protect against the risk of CHD. In addition, we also found significant interactions between TG levels and the three polymorphisms of the AdipoQ gene included in our study.

Our findings are that the AdipoQ gene has dual relations with CHD (depending on the focus of polymorphism studied) – a risk factor for CHD (related to rs3774261G) and a protective role against CHD (related to SNP rs2082940T). AdipoQ (the adiponectin gene) expressed primarily in adipose tissues and vascular tissues as well, encodes the protein adiponectin [47]. Out of the many polymorphisms in the AdipoQ gene, only a small portion of these polymorphisms have been studied in relation to adiponectin, and CVD or CHD with various results being reported, and it is possible that many functional polymorphisms responsible for the observed associations are yet to be investigated [48]. A study among patients with type 2 diabetes by Lacquemant and colleagues on the rs2241766 polymorphism of the AdipoQ gene reported that this polymorphism is associated with increased risk of coronary artery disease in diabetic patients [34] but Bacci and team found that the rs1501299 polymorphism of this same gene is associated decreased coronary artery disease risk [27] – thus creating inconsistency as to the exact role of the AdipoQ in CHD. Most of the studied effects of AdipoQ in relation to many health conditions, including CHD, stroke, diabetes, obesity, myocardial infarction (MI) and other CVDs, are related to the circulating levels and effects of adiponectin – the protein encoded by the AdipoQ gene [33, 44, 49–54]. Studies have shown that hypoadiponectinemia is associated with the prevalence of CVD [55–58], and that high levels of adiponectin are associated with lower risk of CVD [54, 59]. Some studies have reported that certain polymorphisms in the adiponectin coding gene, AdipoQ are strongly associated with adiponectin levels [60–63]. The dual effect of AdipoQ in CHD as found in this present study may be related to the type of AdipoQ polymorphism, which in turn may have direct or indirect effect on the level of circulating adiponectin or other mechanism, and the development of CHD. However, more studies recruiting larger samples and exploring functional roles of these polymorphisms are required to further explain these differences.

The G allele of the rs3774261polymorphism was significantly higher in CHD cases than controls (Table 4) and our inheritance analyses revealed that this polymorphism is associated with increased risk of CHD (Table 5)-suggesting that the G allele is a risk factor for CHD. This finding is concordant with the finding of Zhang et al that the rs2241766G allele and rs266729G allele increase odds of CVD, while the rs1501299T allele decreases the odds of CVD [64]. Two separate Meta–analyses on type 2 diabetes patients by Qi et al [65] and Sun and colleagues [66] revealed that the T allele is protective against CVD risk. Our study found no statistically significant difference in the distribution of the T allele between cases and controls but as a homozygous minor allele, the TT variant was found in the rs2082940T polymorphism of the AdipoQ gene – a polymorphism found in our study to protect against CHD.

The exact mechanism by which variations in the AdipoQ gene contribute to CHD development is not well understood. However, adiponectin (the protein encoded by AdopoQ) has been shown to have anti‑inflammatory effects that play important roles in protecting against atherosclerosis [67]. In mechanically injured arteries, Matsuda and colleagues demonstrated that adiponectin deficiency aggravates neointimal thickening, while adiponectin supplement attenuates this effect, presumably through the suppressive effect of adiponectin on the proliferation and migration of vascular smooth muscle cells [68]. Polymorphism in the AdipoQ gene has been shown to affect plasma levels of adiponectin. A study by Kondo and team demonstrated that plasma adiponectin concentrations of subjects carrying a certain variant of the AdipoQ gene (I164T mutation) were lower than those of subjects without the mutation, and that these subjects showed some features of atherosclerosis among other features of metabolic syndrome [69]. Our present study did not aim to investigate the role of adiponectin in CHD but previous studies have shown that hypoadiponectinemia correlates with CVDs development [70, 71]. Studying the functional properties of the various polymorphisms found in this gene, in particular, how these variants affect plasma levels of adiponectin will shed more light on the role of AdipoQ in CHD and other disease conditions. The rs3774261 polymorphism is intron located – a non-coding region. However, polymorphisms in non-coding regions can affect gene splicing, mRNA degradation, transcription products binding and gene expression. Thus, any of these mechanisms can be possible pathway to explain rs3774261 variant’s association with CHD.

For complex diseases (multifactorial diseases) like CHD, studies have shown that both genetic variations and environmental factors interact to play critical roles in their development [71–73]; and that where interactions do exist, the environmental effects on disease causation will be modified by genotypes. The present study found that the association between any of the SNPs rs3774261, rs1063537 or rs2082940 in the AdipoQ gene and CHD can be influenced by interactions with serum triglycerides. Atherosclerosis is the main cause of coronary artery disease and studies have shown that there is an association between high serum lipids levels and the risks of atherosclerosis and metabolic syndrome in adolescence and adulthood [74, 75]. Studies in South Africans [76] and Europeans [77, 78] revealed that serum lipid level is influenced by genetic variants as well as environmental factors; and variations in the AdipoQ gene is among the genetic factors associated with serum lipid levels. Park and colleagues found that ^_^11377 C/G variant of the AdipoQ gene is significantly associated with total cholesterol and that its GeTeG haplotype carriers had higher LDL cholesterol levels than non-carriers [79]. Our study found no significant interactions with LDL-C, HDL-C, or Total Cholesterol; however triglycerides (found here to interact with AdipoQ gene polymorphisms in the risk of CHD) are major components of very-low-density lipoprotein (VLDL) and chylomicrons, and play an important role as energy sources in metabolism and transporters of dietary fat [80]. Elevated triglycerides blood levels in humans have been linked to atherosclerosis and, by extension, the risk of CHD and other heart diseases [81]. Hence, we are of the opinion that one explanation for our finding that SNP rs3774261G is associated with the risk of CHD could be that this AdipoQ polymorphism can cause variations in serum triglycerides leading to atherosclerosis and subsequently to the development of CHD.

Our study has some limitations, and the first among them is that our study subjects were recruited from two separate hospitals – making selection bias difficult to avoid. Second, our study participants were entirely Northern China’s Han Chinese and differences that might exist between this ethnic group and other ethnic groups or races means interpretations of findings from our study cannot be extended to other populations – thus necessitating the need for replication of our study in other ethnic groups or races. A third limitation is our use of slightly different inclusion criteria for CHD cases and for controls: whereas only electrocardiographic profile and clinical evaluations were used to determine that controls were CHD free, controls were not subjected to coronary angiography examination (the benchmark used for the confirmation of CHD). Asymptomatic CHD cases might have been recruited in the control arm of the study leading to hidden effects.

Despite the limitations of this study, the careful selection of SNPs in the AdipoQ gene leading to both the protective and risk effects of this gene in the development of CHD being revealed in a single study, worth mentioning in the academic literatures. To the best of our knowledge no single study before has reported the dual effects of AdipoQ gene on CHD. Hence, further studying the functions of the rs3774261 and rs2082940 variants of the AdipoQ gene will provide insight into the role of this gene in CHD and thus provide interventions to manage this condition. The finding that the associations between these polymorphisms and CHD interact with serum triglycerides also worth recognition. Lipids play critical roles in the development of CVDs. The rs3774261G SNP as risk factor for CHD (as shown in this study) might be explained by the possibility of this SNP causing adverse variations in blood lipid levels.

## Conclusions

We aimed to investigate the genetic association between the risk of Coronary Heart Disease (CHD) and the rs3774261, rs1063537 and rs2082940 SNPs of AdipoQ gene; and whether there is an interaction between any existing association and environmental factors, in Northeast Han Chinese populations. We found that the polymorphisms in the AdipoQ gene can have dual effects on the development of CHD in Northeast Han Chinese – a protective effect exhibited by the rs2082940 T variant of the gene, and a risk factor for CHD as shown with the rs3774261 G SNP. These findings will help explain that the conflicting reports about the roles of AdipoQ in CHD might be the underlying effects of the various polymorphisms of this gene. This study also revealed that the association between CHD and genetic variations in the AdipoQ gene has interaction with serum triglycerides in our study population. However, further studies recruiting larger samples in different ethnicities or races are needed to further confirm our findings.

## Abbreviations

AdipoQ: adiponectin, C1Q and collagen domain containing
CAD: coronary artery disease
CHD: coronary heart disease
CVDs: cardiovascular diseases
HDL-C: high density lipoprotein cholesterol
LDL-C: low density lipoprotein cholesterol
SNPs: single nucleotide polymorphisms
TC: total cholesterol
TG: triglyceride
